# Pliocene Paleoenvironments of Southeastern Queensland, Australia Inferred from Stable Isotopes of Marsupial Tooth Enamel

**DOI:** 10.1371/journal.pone.0066221

**Published:** 2013-06-12

**Authors:** Shaena Montanari, Julien Louys, Gilbert J. Price

**Affiliations:** 1 American Museum of Natural History, Richard Gilder Graduate School, New York, New York, United States of America; 2 The University of Queensland, School of Earth Sciences, St. Lucia, QLD, Brisbane, Australia; University of New South Wales, Australia

## Abstract

The Chinchilla Local Fauna is a diverse assemblage of both terrestrial and aquatic Pliocene vertebrates from the fluviatile Chinchilla Sand deposits of southeastern Queensland, Australia. It represents one of Australia's few but exceptionally rich Pliocene vertebrate localities, and as such is an important source of paleoecological data concerning Pliocene environmental changes and its effects on ecosystems. Prior inferences about the paleoenvironment of this locality made on the basis of qualitative observations have ranged from grassland to open woodland to wetland. Examination of the carbon and oxygen isotopes in the tooth enamel of marsupials from this site represents a quantitative method for inferring the paleoenvironments and paleoecology of the fossil fauna. Results from Chinchilla show that *Protemnodon* sp. indet. consumed both C3 and C4 photosynthesis plant types (mean δ^13^C = −14.5±2.0‰), and therefore probably occupied a mixed vegetation environment. *Macropus* sp. indet. from Chinchilla also consumed a mixed diet of both C3 and C4 plants, with more of a tendency for C4 plant consumption (mean δ^13^C = −10.3±2.3‰). Interestingly, their isotopic dietary signature is more consistent with tropical and temperate kangaroo communities than the sub-tropical communities found around Chinchilla today. Other genera sampled in this study include the extinct kangaroo *Troposodon* sp. indet. and the fossil diprotodontid *Euryzygoma dunense* each of which appear to have occupied distinct dietary niches. This study suggests that southeastern Queensland hosted a mosaic of tropical forests, wetlands and grasslands during the Pliocene and was much less arid than previously thought.

## Introduction

The Chinchilla Local Fauna of southeastern Queensland represents one of the few well-studied and diverse Pliocene vertebrate assemblages in Australia [1], [2]. The vertebrate assemblage of the Chinchilla Local Fauna, which is derived from the Chinchilla Sand, is represented by an array of fish, reptiles, birds, marsupials, and rodents [2]. Paleoenvironmental reconstructions based on faunal components within the assemblage suggest that a mosaic of habitats occurred around the area during the Pliocene. For instance, the presence of tree kangaroos, koalas and forest wallabies implies the presence of forests [3]–[6]. Large-bodied grazing marsupials suggest the presence of widespread, open grasslands [7]. Numerous aquatic and wetland fossil taxa present in the assemblage imply the occurrence of extensive and permanent water bodies [8]–[11]. Conversely, other information derived from dasyurids suggests seasonally arid climates [12]. It is important to note that interpretations of the Chinchilla Local Fauna's Pliocene paleohabitats are based on qualitative interpretations of gross morphology or taxonomic-based inferences. For example, high-crowned molars of macropodids reflect grazing diets, therefore, paleohabitat interpretations made on the basis of these marsupials suggest the presence of grasslands [7]; presence of extinct fossil birds that may be related to modern water birds have been used to infer the presence of wetlands [11]. Quantitative and geochemical methods of paleoenvironmental reconstructions commonly give more precise interpretations of past habitats, but until now, have not been applied to any Pliocene locality in Australia.

The Pliocene is a critical period for understanding the origins and evolution of Australia's unique modern biota. It is during this time that the Australian fauna first began to take on its modern appearance and distinctiveness, with many modern Australian marsupials, such as the agile wallaby *Macropus gracilis*, first appearing in Pliocene fossil deposits [1]. The Pliocene also documents the first paleobotanical evidence of grasslands [13], which in turn led to the diversification of many marsupial groups through increased use of this resource (e.g., vombatimorphian vombatids (wombats) and macropodids (kangaroos)). Pliocene localities are rare in Australia [14] and it is vital to determine the climate and environment of this time period in order to provide a basis of comparison with the Quaternary, when humans began changing the landscape and ecosystems of the continent in a more direct fashion.

Stable isotope geochemistry of fossil vertebrate tooth enamel is a well known method of discerning paleoecology, paleoenvironments, and paleoclimates e.g. [15]–[17]. The carbon contained in plants consumed by herbivores is incorporated in tooth enamel and does not change during the life of the animal once incorporated [18]. Oxygen incorporated in tooth enamel comes from an animal's body water, which in turn is largely reflective of drinking water composition [19]. Stable isotope geochemistry methods have been shown to be useful in reconstructing diets and environments of marsupials from the Quaternary of Australia [20]–[22] but have not yet been applied to fossils from the Chinchilla Local Fauna. Our study represents the first application of quantitative paleoecological techniques to this region, and will allow for the reconstruction of the environments present in southeastern Queensland during the Pliocene. Our analyses will also allow us to develop better insights into the diets and niche partioning of Pliocene fossil marsupials. In this study we address the following questions:

- What is the geochemical evidence for pervasive Pliocene grasslands within faunal elements derived from the Chinchilla Sand?
- How does the Pliocene climate of the region compare to modern times?
- Is there evidence for dietary niche differentiation between taxa?

### Geology and age of formation

The name Chinchilla Sand was originally proposed by Woods (1960) for the predominantly sandy-clayey sequence of fluviatile sediments exposed in the Condamine River and nearby gulley systems. The formation spans a distance of roughly 65 km from Nangram Lagoon, situated about 20 km northeast of Condamine, in the west and Warra in the east. The sediments are generally weakly consolidated, with clasts ranging in size from clay to pebbles, although the dominant lithology is sandy. Local lithification occurs as a result of calcium carbonate or iron oxide [23]. The quartzite material, including silcrete and ferruginous sandstone, are interpreted to be derived from the Mesozoic Orallo Formation and its lateritized profiles [24]. The Chinchilla Sand is thought to reach a maximum thickness of approximately 30 m, on the basis of pits and wells sunk near Brigalow [23]. It is overlain unconformably by dark alluvial Quaternary clays and sands [24].

Vertebrate fossils are found throughout the Chinchilla Sand lithological units. Biocorrelation of the Chinchilla Local Fauna with the paleomagnetically dated Kanunka and Toolapinna Local Faunas of central Australia suggests an age of approximately 3.4 Ma [25]. ‘Stage of evolution’ comparisons based on marsupials suggests that the Chinchilla Local Fauna postdates the Bluff Downs Local Fauna of northeastern Australia, which itself has been given a minimum age of 3.6 Ma [26]. Thus, the Chinchilla Local Fauna mostly likely dates to the early Piacenzian. Direct dating of this site and more detailed stratigraphic analyses are currently in preparation.

### Spread of grassland in Australia

During much of the first half of the Miocene, forests were widespread throughout Queensland [27], with more closed habitat conditions found more generally throughout the continent [13]. It was not until the late Miocene that enhanced aridity led to the contraction of forests and expansion of open habitats [13]. Central Australia became dry with open woodland and chenopod shrub dominated landscape. Although the Pliocene began with warm, wet conditions, allowing the re-expansion of *Nothofagus* and other rainforest flora [13], it soon began to dry again.

The first paleobotanical evidence of grasslands in Australia appears in the form of desert chenopod shrub phytoliths in northwestern Australia during the Pliocene [13]. It has been thought that this represents the first major spread of grasslands, a hypothesis supported by the increase in grazing animals at the same time [28]. Additionally, evidence of phytoliths in oceanic cores on the Lord Howe Rise off the eastern coast that show there was a spread of grasslands on the eastern side of the continent at the same time [29]. Marine and pollen records illustrate a trend towards open woodland and grassland environments during the Pliocene, but there was still considerably higher rainfall than today [13]. Wet sclerophyll forests became common near the eastern, southeastern and northwestern coastal regions [30], [31], with drier forests and woodlands present further inland [32]. Although rainforests persisted in eastern Australia during the Pliocene, the rise of herbaceous taxa during this time is correlated with increased seasonality [33]. By examining stable isotope geochemistry we can determine the proportions of grasslands and forests that were present during the Pliocene in southeastern Queensland, and compare those values with modern conditions in both tropical and subtropical zones to determine the most likely conditions present during that time.

### Stable isotope ecology of mammals

#### Carbon isotopes

The carbon (δ^13^C) found in the carbonate phase of bioapatite is related to the δ^13^C of ingested organic material [15]. The different photosynthetic pathways, C3 (Calvin-Benson) and C4 (Hatch-Slack), are characterized by different δ^13^C values and this is in turn reflected in the tooth enamel of mammalian herbivores. The carbon isotope ratios of plants change depending on their photosynthetic pathway and environmental conditions [34]. C3 plants have a δ^13^C ranging from −32‰ in understory canopy conditions to −21‰ in drier environments [35]. Generally, the δ^13^C of C3 plants increases as the climate gets drier. C4 plants, which are mainly grasses, can range from −15 to −9‰. C3 plants dominate cool, moist regimes. In Australia, abundances of C3 plants decline with increasing temperature and/or decreasing spring rains, while C4 grass is most abundant in areas where summer is hot and wet [36].

The isotopic fractionation between food (diet) and tissue (tooth enamel) has been studied in a variety of mammalian test systems. The fractionation constant between bulk diet and δ^13^C of tooth enamel in wild herbivores is between +9 and 12‰ [37]–[39]. However, in more recent studies of marsupials, a ∼12‰ fractionation between diet and enamel δ^13^C was found in kangaroos and wombats [21], [40]. This fractionation was used to examine diets of Pleistocene macropodids in Forbes et al. (2010) and will be used in this study.

It is also important to take into account the effect of weaning on the δ^13^C composition of tooth enamel. Early formed molars are ^13^C depleted compared to late formed molars [22], [40], due to the shift from milk to solid food in the diet of marsupials. This is either because of a change in internal physiological fractionation in the animal, or because the milk has more low δ^13^C fat than plant fodder [40]. Currently, this weaning effect has only been tested in large macropodids [40].

#### Oxygen isotopes

Oxygen isotopes in water vary due to temperature, evaporation, and source of air masses [41]. Terrestrial vertebrates do not directly ingest precipitation; instead, their water is primarily ingested from streams, ponds, lakes, and leaves. Each of those reservoirs typically has different δ^18^O relative to precipitation, due to preferential incorporation of the ^18^O isotope into condensate during evaporation. The δ^18^O of organisms with body water composed mainly of drinking water can be used to reconstruct the landscape hydrology in paleoenvironments (e.g. [42]).

Animals such as modern day kangaroos have low drinking water requirements, so the δ^18^O of their tooth enamel mainly reflects that of leaf water (from food) and therefore, relative humidity [43]. Plant leaf water is subject to evaporative enrichment of the heavy isotope ^18^O at low humidity [44], and this δ^18^O signature is passed on to the animals that consume these leaves, so it is possible fossil herbivores can be used as a paleohumidity proxy. Murphy (2007b) examined sources of δ^18^O variation in kangaroo (*Macropus* spp.) tooth enamel. Relative humidity explained a large proportion of the δ^18^O tooth enamel variance, but they also uncovered a previously unreported correlation between mean annual temperature and relative humidity. Therefore, they recommended not using δ^18^O of fossil teeth in herbivores to reconstruct relative humidity unless there is a reliable estimate of air temperature at the same locality [45]. Additionally, they also found no effect from weaning on the δ^18^O of molars within individuals.

## Methods

### Collection

**Figure 1:**
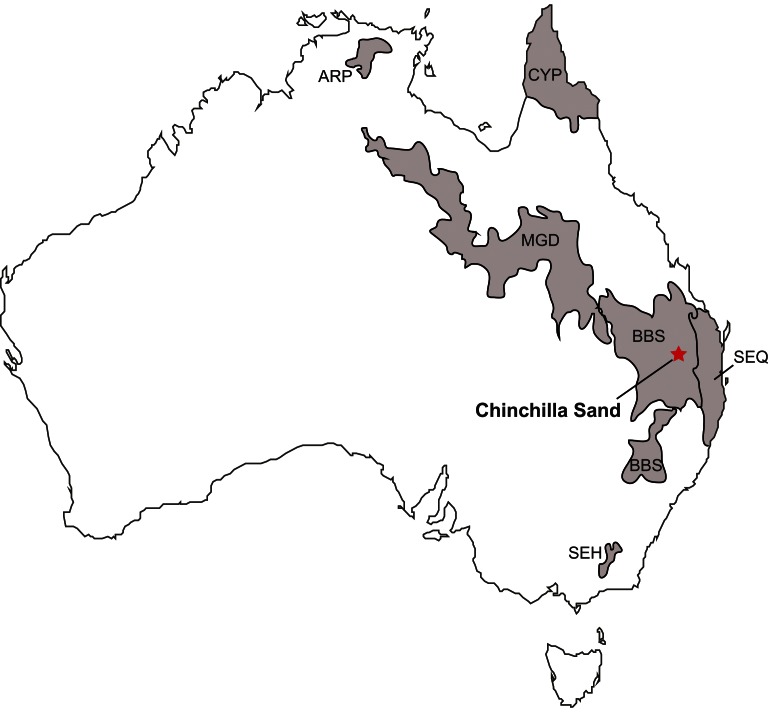
Map of Chinchilla Sand Formation fossil locality. Chinchilla is marked on this map, along with the shaded areas representing the biogeographic zones where modern kangaroo tooth enamel stable isotope values were taken from Murphy et al. 2007a to compare to fossil values. Abbreviations for biogeographic zones are in the methods section.

Fossils were collected from one of the Chinchilla Sand Formation localities, the Chinchilla Rifle Range, in Chinchilla, Queensland (Figure 1) by Ces and Doris Wilkinson over a period of more than 20 years. Such fossils were subsequently donated to the Queensland Museum. Most were recovered as surface finds uncovered by erosion of unconsolidated sediments in the main gully system; however, some were excavated from Dig Site. No permits were required for the described study, which complied with all relevant regulations. Permission for sampling of the fossils was provided by the Queensland Museum. Fossils were loaned and returned following isotopic analysis. All the fossils examined herein were recovered from the Chinchilla Sand. In this study, we chose to perform stable isotope analysis of the tooth enamel of four sympatric vertebrates: macropodids *Protemnodon* sp. indet., *Troposodon* sp. indet., and *Macropus* sp. indet. and diprotodontid *Euryzygoma dunense*. Not every specimen sampled could be identified to species level, which could indicate that we sampled a variety of species within each genus. While this limits our ability to make dietary attributions to specific species, the conclusions we can draw from genus-level molar identification reveal important new information about the range of environments at the locality. We chose these taxa on the basis of their abundant availability in the fossil collections. We hypothesize that sampling four large bodied herbivorous marsupials from the same habitat will give a clear snapshot of most available consumable plant fodder at a site.

### Stable isotopes

Bulk samples of enamel were obtained by using a Dremel drill to remove a flake of enamel, which was subsequently ground into fine powder using a ceramic mortar and pestle. For bioapatite samples, over 1000 µg was used to obtain an accurate result. Powdered samples of bioapatite were subsequently treated using 30% H_2_O_2_ and 0.1 N acetic acid to remove organic material and surficial carbonates [46]. Analyses were run on a Thermo Electron Corporation Finnegan Delta plus XP mass spectrometer in continuous-flow mode via the Thermo Electron Gas Bench peripheral and a GC-PAL autosampler housed at the University of Rochester. Carbon and oxygen isotopic results are reported in per mil (‰) relative to VPDB (Vienna Pee-Dee Belemnite) with an allowable 2-sigma uncertainty of 0.12‰ and 0.20‰ for carbon and oxygen respectively. Statistical analyses, ANOVA and Tukey HSD, were all performed on Microsoft Excel 2011 and PAST ver. 2.14.

Isotopic ratios of carbon are expressed using the permil notation, such as: δ^13^C (permil, ‰) = ((R_sample_/R_standard_-1)×1000), where R = ratio of ^13^C/^12^C of an unknown sample relative to a known standard VPDB [45]. Oxygen isotopes are expressed similarly to carbon isotopes: δ^18^O (permil, ‰) = ((R_sample_/R_standard_-1)×1000), where R = ratio of ^18^O/^16^O of an unknown sample relative to a known standard, either VPDB or V-SMOW [47]. In this paper, oxygen isotopes are reported with respect to VPDB.

It was previously mentioned that δ^13^C of enamel can change due to weaning in marsupials. Such ontogenetic changes must be taken into account when performing a study on fossil marsupials, so in our study we used only the third or fourth molars (the last erupting teeth) in our analysis wherever possible, so the δ^13^C signal we interpreted was most likely from plant diet, not milk diet [40]. Modern stable isotope data from *Macropus* spp. from around Australia was obtained from Murphy et al. (2007a). Isotopic data from *Macropus* species sampled in Murphy et al. (2007a) and included in this study were *M. giganteus*, *M. rufus*, *M. fuliginosis*, *M. robustus*, *M. rufogriseus*, *M. agilis*, *M.antilopinus*, and *M. bernardus*. It is vital to note that there is a ∼1.2 ‰ depletion in δ^13^C in modern samples compared to pre-industrial δ^13^C CO_2_ of atmosphere due to the burning of fossil fuels (known as the Suess Effect) [48], [49]. We therefore corrected for this enrichment by applying a correction of −1.2‰ to all Pliocene samples in order to allow for comparisons between carbon isotopes of modern and fossil marsupial tooth enamel [50]. We used specimens of *Macropus* spp. from Murphy et al. (2007a) that came from the following biogeographic regions noted in their supplementary information: CYP (Cape York Peninsula), ARP (Arnhem Plateau), BBS (Brigalow Belt South), and SEQ (South East Queensland), SEH (South Eastern Highlands) and MGD (Miller Grass Downs). We chose kangaroos from these regions because they encompass major modern climates we wish to compare to Chinchilla Sand. CYP and ARP are classified as tropical, BBS and SEQ are subtropical, SEH is temperate, and MGD is grassland/desert. Regions are defined on the basis of Interim Biogeographic Regionalisation for Australia version 7 [51]. Climates of Australia are based on a Koppen classification system from the Australian Bureau of Meterology [52].

## Results

### Carbon isotopes

Means and standard deviation of isotopes in each taxon group are presented in Table 1. The overall range of δ^13^C means over all taxa is −14.5 to −10.3‰, which corresponds to a diet of −26.5 to −22.3‰ when the ∼12‰ enrichment is accounted for. The range of modern *Macropus* spp. δ^13^C of enamel in the same region as Chinchilla, taken from Murphy et al. (2007a), is −14.1 to −2.2‰, corresponding to a diet of −26.1 to −15.7‰. ANOVA shows significant differences in δ^13^C between the four fossil taxa analyzed at Chinchilla (p<0.001). For the comparisons between modern and fossil kangaroo *Macropus* spp. samples, δ^13^C was different between the six tropical, subtropical, temperate, and desert zones and the fossil Chinchilla locality (p<0.001). ANOVA results are summarized in Table 2.

**Table 1:** Stable isotope general statistics.

Taxon	n	δ^13^C	δ^13^C Suess effect	stdev	δ^18^O	stdev
*Euryzygoma dunense*	12	−11.1	−12.3	2.8	−0.2	1.4
*Macropus* sp. indet.	24	−9.1	−10.3	2.3	−1.5	1.9
*Protemnodon* sp. indet.	8	−13.3	−14.5	2.0	−2.6	2.4
*Troposodon* sp. indet.	6	−11.6	−12.8	2.5	−1.5	1.0

Mean, n, and standard deviation (stdev) for both carbon and oxygen isotope values for all materials sampled. δ^13^C Suess effect is the raw carbon isotope value with 1.2 per mil subtracted to account for the modern depletion in atmospheric δ^13^C. Isotope values are presented in per mil (‰).

**Table 2:** Summary of ANOVA results.

Variable	dF	F	p	significant
δ^13^C fossils only	3	6.919	0.0006099	yes
δ^13^C modern and Chinchilla *Macropus* spp.	6	54.5	1.72E-33	yes
δ^18^O fossils only	3	2.788	0.05108	no
δ^18^O modern and Chinchilla *Macropus* spp.	6	52.12	1.37E-32	yes

Summary of the test statistics for each ANOVA, including degrees of freedom (dF), F-statistic, p (probability), and significance.

The results of Tukey's HSD test from the carbon isotope ANOVAs are in Table 3. *Protemnodon* sp. indet. δ^13^C is significantly different than that of *Macropus* sp. indet., but is not differentiated from the δ^13^C of *Troposodon* sp. indet. or *Euryzygoma dunense*. *Macropus* sp. indet. only shows differences from *Protemnodon* sp. indet.; there is no statistical difference between *Macropus* sp. indet. and *E. dunense* or *Macropus* sp. indet. and *Troposodon* sp. indet..

**Table 3:** Summary of results from Tukey's HSD test from the δ^13^C and δ^18^O fossil ANOVAs.

δ^13^C	*Macropus* sp. indet.	*Protemnodon* sp. indet.	*Troposodon* sp. indet.
*Euryzygoma dunense*	0.2551	0.236	0.9759
*Macropus* sp. indet.		**0.002533**	0.1168
*Protemnodon* sp. indet.			0.4451
δ^18^O			.
*Euryzygoma dunense*	0.4172	**0.03481**	0.4397
*Macropus* sp. indet.		0.5884	1
*Protemnodon* sp. indet.			0.5641

Comparisons are pairwise and p values are in bold if significant (p = 0.05).

When fossil Chinchilla *Macropus* sp. indet. are compared to modern *Macropus* spp. from six different biogeographic regions of Australia using ANOVA, δ^13^C is significantly different (Table 2). The *Macropus* spp. from the modern region that contains the Chinchilla locality and the surrounding area, BBS and SEQ, have δ^13^C values significantly higher than fossil *Macropus* sp. indet. when examined with Tukey's HSD test (Table 4). *Macropus* spp. from MGD are similar to *Macropus* spp. from all regions, including Chinchilla. On the other hand, there was no significant difference between fossil *Macropus* sp. indet. δ^13^C from Chinchilla and modern *Macropus* spp. from tropical regions CYP and ARP.

**Table 4:** Summary of results from Tukey's HSD test from the δ^13^C and δ^18^O ANOVAs of modern *Macropus* spp. and fossil *Macropus* sp. indet. from Chinchilla.

δ^13^C	BBS	CYP	ARP	MGD	SEH	Chinchilla
SEQ	0.5603	**3.89E-05**	**9.06E-05**	0.202	**2.57E-05**	**2.57E-05**
BBS		**0.02532**	0.06355	0.9965	**2.57E-05**	**2.60E-05**
CYP			0.9999	0.1371	**2.57E-05**	0.1927
ARP				0.2668	**2.57E-05**	0.09244
MGD					**2.57E-05**	**3.74E-05**
SEH						**0.0004263**
δ^18^O						
SEQ	**0.0008475**	0.9964	0.06652	**2.57E-05**	**0.001761**	0.1508
BBS		**7.36E-05**	**2.57E-05**	0.4295	**2.57E-05**	**2.57E-05**
CYP			0.277	**2.57E-05**	**0.01669**	0.4729
ARP				**2.57E-05**	0.9375	0.9999
MGD					**2.57E-05**	**2.57E-05**
SEH						0.8069

Comparisons are pairwise and p values are bolded if significant (p = 0.05). Regional abbreviations: CYP (Cape York Peninsula), ARP (Arnhem Plateau), BBS (Brigalow Belt South), and SEQ (South East Queensland), SEH (South Eastern Highlands) and MGD (Miller Grass Downs).

### Oxygen isotopes

Means and standard deviations of δ^18^O from sample fossil taxa are in Table 1. The values of fossil taxa sampled range from −2.6 to −0.2‰. Modern *Macropus* spp. from the BBS region containing Chinchilla have an enamel δ^18^O range from −2.8 to 4.2‰. There is not a wide variation in the δ^18^O between taxa; ANOVA of δ^18^O values shows no significant differences between the four fossil genera (p = 0.051). The ANOVA between δ^18^O of modern *Macropus* spp. from the six biogeographic zones compared with δ^18^O from fossil Chinchilla *Macropus* sp. indet. show a significant difference. ANOVA results are summarized in Table 2.

The results of Tukey's HSD test from the oxygen ANOVAs are contained in Table 3. There is a significant difference in the pairwise comparisons between *Protemnodon* sp. indet. and *E. dunense.* When Chinchilla fossil *Macropus* sp. indet. δ^18^O are compared to δ^18^O of *Macropus* spp. from subtropical (BBS, SEQ), tropical (ARP, CYP), temperate (SEH), and grassland (MGD) regions, Chinchilla fossil *Macropus* sp. indet. are only different from values from BBS and MGD; BBS is the region that contains modern-day Chinchilla (Table 4).

## Discussion

### Dietary niches

**Figure 2:**
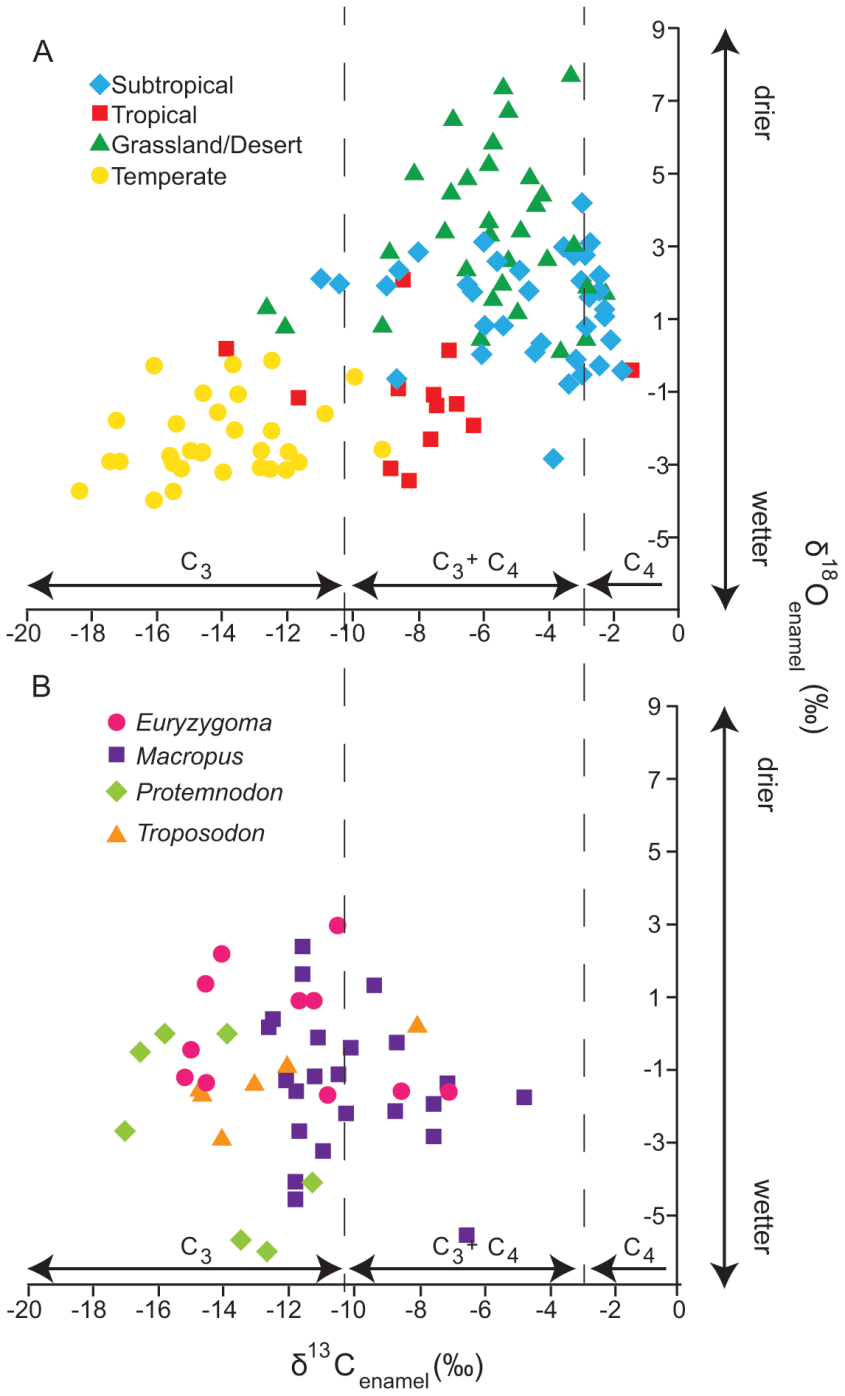
Bivariate plot of carbon and oxygen for fossil and modern teeth. A. δ^18^O vs. δ^13^C values for *Macropus* spp. from six modern localities, grouped by their climatic region. Labels on axes indicate the boundary between a C3 dominated and a C3/C4 mixed environment. B. δ^18^O vs. δ^13^C values for *Troposodon* sp. indet., *Protemnodon* sp. indet., *Euryzygoma dunense*, and *Macropus* sp. indet. from Chinchilla Sand. Each taxon is marked by a symbol as seen in the legend.

Between the four fossil taxa sampled here, we observe clear indications of unique dietary niche separation (Figure 2). *E. dunense*, *Macropus* sp. indet., and *Troposodon* sp. indet. consumed a mixed C3 and C4 diet, with average δ^13^C_diet_ = −24.3‰, −22.3‰ and −24.8‰ respectively (Table 5). These three taxa consumed a mixed diet, but the majority of it was comprised of C3 plants; the percentage of C3 plants in the diet was calculated using equation (1) in Johnson et al. (1997) [53]. *E. dunense* had a δ^13^C_diet_ that indicates it primarily fed on C3 plants, which is in concordance with a previous estimate of diet from another diprotodontid, *Diprotodon* [54]. *Protemnodon* sp. indet., thought to be a forest-dwelling marsupial based on morphological evidence [4], unmistakably occupied a different niche than the other three taxa, based on the fact it has the most negative mean δ^13^C out of all four taxa sampled (−14.5‰). Our results indicate that *Protemnodon* could have subsisted primarily on C3 browse, such as would be found in a sclerophyll forest. Overall, there is evidence of a C4 grass signature in the diets of these animals, but C3 plants comprise the majority.

**Table 5:** δ^13^C diet and %C3 diet of fossil and modern marsupials.

Taxon	Locality	δ^13^C diet	% C3 diet
*Euryzygoma* sp. indet.	Chinchilla	−24.3	84.3
*Macropus* sp. indet. Fossil	Chinchilla	−22.3	70.0
*Protemnodon* sp. indet.	Chinchilla	−26.5	100.0
*Troposodon* sp. indet.	Chinchilla	−24.8	87.9
*Macropus* spp. Modern	SEQ	−15.7	22.8
*Macropus* spp. Modern	BBS	−17.3	34.2
*Macropus* spp. Modern	CYP	−20.1	54.4
*Macropus* spp. Modern	ARP	−19.8	52.4
*Macropus* spp. Modern	MGD	−17.8	38.1
*Macropus* spp. Modern	SEH	−26.1	96.9

δ^13^C diet is obtained by taking the average δ^13^C of enamel and subtracting the diet-enamel enrichment factor of 12‰ (Fraser et al. 2008). %C3 diet is calculated using equation 1 in Johnson et al. (1997) with 26.5‰ and 12.5‰ used as the average for C3 and C4 plants in the landscape.

Due to the δ^18^O of the fossil Chinchilla taxa not being high due to enrichment in ^18^O, the environment was most likely mesic. This not only indicates an environment with moderate to high rainfall, but when combined with the carbon isotope data, also indicates that C3 plants were more likely present in forests rather than grasslands, as grasses are prevalent in drier environments. Other evidence, such as the relatedness of taxa sampled like *Protemnodon* sp. indet. to other forest wallaby browsers, indicates the C3 plants consumed in this environment were most likely trees in an open forest [4]. It appears that, although C4 grasslands had spread to this region, grass was not the primarily dietary intake for any of these four taxa. Oxygen isotopes between the four taxa show no statistical differences, and we hypothesize that this is a result of drinking water from frequently replenished water sources that were connected without much evaporation.

### Paleoenvironment of the Chinchilla Sand Formation

To better understand the paleoenvironment of the Chinchilla Sand fossil locality, it is useful to compare our results to modern day signatures found in *Macropus* spp., which show how δ^13^C and δ^18^O naturally vary in a known landscape. The diet of *Macropus* spp. in the modern region of Queensland around Chinchilla is statistically different from all of the δ^13^C signatures in the tooth enamel of the Pliocene marsupials. It is apparent that the diets of kangaroos in this region today are dominated by C4 grasses with highly positive δ^13^C values (Table 6). This suggests that the proportion of C4 grasses in the landscape today is far greater in this region than they were during the Pliocene.

**Table 6:** Summary of general statistics for modern *Macropus* spp. values in different biogeographic and climatic regions from Murphy et al. (2007a).

Taxon	n	Region	Climate	δ^13^C	stdev	δ^18^O	stdev
*Macropus* spp.	14	SEQ	Subtropical	−2.2	1.9	−0.1	1
*Macropus* spp.	24	BBS	Subtropical	−3.8	2.6	2.3	0.8
*Macropus* spp.	9	CYP	Tropical	−8.1	3.1	−0.4	1.1
*Macropus* spp.	6	ARP	Tropical	−7.8	0.9	−1.7	1.6
*Macropus* spp.	32	MGD	Grassland/Desert	−5.8	2.4	3.4	2.1
*Macropus* spp.	31	SEH	Temperate	−14.1	2.2	−2.3	1.1

Isotope values are presented in per mil (‰). See methods for acronyms of region names. Header labels are taxon, n (sample size), biogeographic region (region), climate, carbon isotope value (δ^13^C), oxygen isotope value (δ^18^O), and standard deviation (stdev).

When examining δ^18^O in addition to δ^13^C, there is a significant difference between the oxygen isotopes of fossil Chinchilla marsupials and modern day *Macropus* spp. from the BBS region. It appears that, out of the two biogeographic zones in this area of Queensland, Pliocene fossil Chinchilla taxa are more similar to that of the modern SEQ zone than the BBS zone. When comparing the δ^13^C of *Macropus* spp. found in tropical biogeographic zones ARP and CYP, the Chinchilla fossil *Macropus* sp. indet. are indistinguishable. The same pattern holds with the δ^18^O; at Chinchilla the δ^18^O of *Macropus* sp. indet. tooth enamel is most similar to that in the tropical regions, SEQ and the temperate SEH. The fact that fossil Chinchilla *Macropus* sp. indet. are so similar to *Macropus* spp. from SEH in δ^18^O could indicate a similar hydrologic regime, and therefore a similar plant structure of C3 forests. It can be useful to compare areas of modern average rainfall with fossil values to get an indication of what paleorainfall could have been [20]. Average rainfall in Chinchilla (BBS) region today is 600–800 mm, while SEQ has a range from 600 up to 1200 mm in small patches near the coast. The Miller Grass Downs (MGD) has 200–500 mm of rainfall per year. In contrast, the CYP and ARP regions receive 1000–2000 mm of rain per year. SEH in southeastern Australia can have mean annual precipitation ranging from 500 up to 1600 mm per year. It is clear that fossil Chinchilla *Macropus* sp. indet. group with those from CYP and ARP, tropical regions of Australia, in both carbon and oxygen values (Figure 2). This suggests that rainfall at the Pliocene Chinchilla was much higher than it is today, and that it was possibly closer to a tropical level of rainfall (over 1000 mm). It also suggests the environment at the locality was significantly more forested due to its dissimilarity from grassland environments sampled (MGD). It is important to remember these are only qualitative indications of paleorainfall and are merely loose estimates based on comparison with modern values; more quantitative work is needed for more precise estimates.

It is difficult to determine the precise mean annual rainfall during the Pliocene based on these results because there is a possibility that the δ^18^O of precipitation was significantly different than it is today. But, using our combined evidence, it appears that Chinchilla in the Pliocene represented a mosaic environment that included mostly forest but also mixed C3/C4 grassland. There is no specific isotopic evidence of a closed canopy, but the dietary signature of the browser *Protemnodon* sp. indet. and the presence of three species of phascolarctids [6] indicates that this could have been present. The presence of many aquatic taxa, such as ducks, pelicans, turtles, lungfish and crocodiles, indicates the presence of extensive long-term water bodies in the region, while the thick fluviatile deposits indicate extensive river systems [2]. Also, our results do not preclude the reconstruction of the Chinchilla paleoenvironment as riparian forests surrounded by tropical grasslands. Our results suggest that tropical conditions, that today are restricted only to northern Queensland and the Northern Territory, could have extended significantly southwards through Queensland during the Pliocene, but further isotopic sampling on a greater range of taxa is needed to confirm this. Although C4 grasslands were spreading across Australia at this time, our results suggest they were not the primary habitat type present in this locality.

## Conclusions

Despite the fact the Pliocene marks the spread of grasses around Australia [13], the depositional area of the Chinchilla Sand was not dominated by C4 grasslands. Instead, the environment was more mixed, with a clear indication of abundant C3 plants, potentially a wet tropical sclerophyll forest. The Pliocene *Macropus* sp. indet. at Chinchilla consumed both C3 and C4 plants. The proportion of C4 grasses in their diets may be confirmed in the future through dental microwear analyses. Both *Euryzygoma dunense* and *Troposodon* sp. indet. were mixed feeders with a tendency towards C3 plants, while the confirmed forest wallaby *Protemnodon* sp. indet. subsisted almost entirely on C3 plants, indicating the likely presence of trees. These inferences are confirmed by our comparison of fossil isotopic values with those of modern *Macropus* spp. from different regions of Australia. We reconstruct the Chinchilla Sand fossil locality as significantly wetter and more vegetated during the Pliocene than today, potentially representing an environment with forests in addition to tropical grasslands and wetlands. Further exploration at this site and neighboring Pleistocene localities will give us a better indication of paleoecology and shifts in paleoenvironments in relation to climate change in the region.

## Supporting Information

Table S1 **Carbon and oxygen stable isotope values.** Raw stable isotope data of Pliocene fossil tooth enamel used in this chapter. Data are presented in per mil (‰).
(DOCX)

Table S2 **Carbon and oxygen stable isotope values.** The stable isotope values used in this paper of modern *Macropus* tooth enamel obtained from Murphy et al. (2007a).
(DOCX)
