# Supplementary material for: Pliocene Paleoenvironments of Southeastern Queensland, Australia Inferred from Stable Isotopes of Marsupial Tooth Enamel
Source: PLoS One. 2013 Jun 12;8(6):e66221. doi: 10.1371/journal.pone.0066221 (PMC3680432; doi:10.1371/journal.pone.0066221)
Supplement: Table S1 — Carbon and oxygen stable isotope values. Raw stable isotope data of Pliocene fossil tooth enamel used in this chapter. Data are presented in per mil (‰). (DOCX) [file pone.0066221.s001.docx]

| Sample Number | Taxon | δ^13^C enamel | with Suess effect (add -1.2) | δ^13^C diet (add -12) | δ^18^O (VPDB) | QM Specimen Number (QMF) |
| --- | --- | --- | --- | --- | --- | --- |
| 1 | *Euryzygoma dunense* | -7.3 | -8.5 | -20.5 | -1.7 | 44968 |
| 2 | *Euryzygoma dunense* | -5.9 | -7.1 | -19.1 | -1.6 | 44969 |
| 3 | *Euryzygoma dunense* | -10.3 | -11.53 | -23.53 | 0.8 | 44967 |
| 4 | *Euryzygoma dunense* | -10.1 | -11.3 | -23.3 | 0.8 | 57151 |
| 5 | *Euryzygoma dunense* | -13.8 | -15 | -27 | -0.5 | 57152 |
| 6 | *Euryzygoma dunense* | -14 | -15.2 | -27.2 | -1.3 | 57153 |
| 7 | *Euryzygoma dunense* | -9.6 | -10.8 | -22.8 | -1.8 | 57154 |
| 8 | *Euryzygoma dunense* | -13.5 | -14.7 | -26.7 | -1.5 | 57155 |
| 9 | *Euryzygoma dunense* | -13.6 | -14.8 | -26.8 | -1.8 | 57156 |
| 10 | *Euryzygoma dunense* | -13.4 | -14.6 | -26.6 | 1.3 | 57150 |
| 11 | *Euryzygoma dunense* | -9.3 | -10.5 | -22.5 | 2.9 | 57149 |
| 12 | *Euryzygoma dunense* | -12.9 | -14.1 | -26.1 | 2.1 | 57148 |
| 13 | *Macropus* sp. indet. | -10.6 | -11.8 | -23.8 | -4.6 | 44978 |
| 14 | *Macropus* sp. indet. | -10.6 | -11.8 | -23.8 | -4.2 | 44986 |
| 15 | *Macropus* sp. indet. | -5.3 | -6.5 | -18.5 | -5.6 | 44987 |
| 16 | *Macropus* sp. indet. | -13.6 | -14.8 | -26.8 | -1.9 | 44988 |
| 17 | *Macropus* sp. indet. | -10.5 | -11.7 | -23.7 | -2.8 | 44990 |
| 18 | *Macropus* sp. indet. | -10 | -11.2 | -23.2 | -1.3 | 44997 |
| 19 | *Macropus* sp. indet. | -3.5 | -4.7 | -16.7 | -1.9 | 44998 |
| 20 | *Macropus* sp. indet. | -5.9 | -7.1 | -19.1 | -1.5 | 55464 |
| 21 | *Macropus* sp. indet. | -6.3 | -7.5 | -19.5 | -2.0 | 55465 |
| 22 | *Macropus* sp. indet. | -8.9 | -10.1 | -22.1 | -0.5 | 55466 |
| 23 | *Macropus* sp. indet. | -6.3 | -7.5 | -19.5 | -2.9 | 55469 |
| 24 | *Macropus* sp. indet. | -7.5 | -8.7 | -20.7 | -2.2 | 55471 |
| 25 | *Macropus* sp. indet. | -9.0 | -10.22 | -22.22 | -2.3 | 44975 |
| 26 | *Macropus* sp. indet. | -10.4 | -11.59 | -23.59 | 1.5 | 44976 |
| 27 | *Macropus* sp. indet. | -9.7 | -10.94 | -22.94 | -3.3 | 44977 |
| 28 | *Macropus* sp. indet. | -11.5 | -12.67 | -24.67 | 0.1 | 44979 |
| 29 | *Macropus* sp. indet. | -9.9 | -11.13 | -23.13 | -0.2 | 44980 |
| 30 | *Macropus* sp. indet. | -9.3 | -10.47 | -22.47 | -1.2 | 44989 |
| 31 | *Macropus* sp. indet. | -10.6 | -11.8 | -23.8 | -1.7 | 44995 |
| 32 | *Macropus* sp. indet. | -10.9 | -12.1 | -24.1 | -1.4 | 44996 |
| 33 | *Macropus* sp. indet. | -11.4 | -12.55 | -24.55 | 0.3 | 44999 |
| 34 | *Macropus* sp. indet. | -7.5 | -8.67 | -20.67 | -0.3 | 45000 |
| 35 | *Macropus* sp. indet. | -8.1 | -9.34 | -21.34 | 1.2 | 55467 |
| 36 | *Macropus* sp. indet. | -10.4 | -11.61 | -23.61 | 2.3 | 55470 |
| 37 | *Protemnodon chinchillensis* | -15.4 | -16.61 | -28.61 | -0.5 | 44993 |
| 38 | *Protemnodon* sp. indet. | -11.5 | -12.7 | -24.7 | -6.0 | 44991 |
| 39 | *Protemnodon* sp. indet. | -10.1 | -11.3 | -23.3 | -4.1 | 44992 |
| 40 | *Protemnodon* sp. indet. | -12.3 | -13.5 | -25.5 | -5.7 | 44994 |
| 41 | *Protemnodon* sp. indet. | -13.5 | -14.65 | -26.65 | -1.7 | 44981 |
| 42 | *Protemnodon* sp. indet. | -14.7 | -15.88 | -27.88 | 0.0 | 44983 |
| 43 | *Protemnodon* sp. indet. | -12.7 | -13.89 | -25.89 | -0.1 | 44984 |
| 44 | *Protemnodon* sp. indet. | -15.9 | -17.1 | -29.1 | -2.7 | 44985 |
| 45 | *Troposodon minor* | -11.9 | -13.1 | -25.1 | -1.5 | 44970 |
| 46 | *Troposodon minor* | -6.9 | -8.08 | -20.08 | 0.1 | 44972 |
| 47 | *Troposodon minor* | -10.9 | -12.06 | -24.06 | -1.0 | 44973 |
| 48 | *Troposodon minor* | -13.5 | -14.71 | -26.71 | -1.7 | 44974 |
| 49 | *Troposodon* sp. indet. | -12.9 | -14.1 | -26.1 | -3 | 44971 |
| 50 | *Troposodon* sp. indet. | -13.5 | -14.72 | -26.72 | -1.8 | 44982 |
