# Supplementary material for: Pliocene Paleoenvironments of Southeastern Queensland, Australia Inferred from Stable Isotopes of Marsupial Tooth Enamel
Source: PLoS One. 2013 Jun 12;8(6):e66221. doi: 10.1371/journal.pone.0066221 (PMC3680432; doi:10.1371/journal.pone.0066221)
Supplement: Table S2 — Carbon and oxygen stable isotope values. The stable isotope values used in this paper of modern Macropus tooth enamel obtained from Murphy et al. (2007a). (DOCX) [file pone.0066221.s002.docx]

| Specimen number | Species | δ^13^C enamel | δ^18^O (VPDB) | Region | δ^13^C diet (add -12) |
| --- | --- | --- | --- | --- | --- |
| S664 | *M. giganteus* | -3.17 | -0.14 | SEQ | -15.17 |
| S665 | *M. giganteus* | -8.68 | -0.75 | SEQ | -20.68 |
| S666 | *M. giganteus* | -3.35 | -0.78 | SEQ | -15.35 |
| S667 | *M. giganteus* | -4.27 | 0.24 | SEQ | -16.27 |
| S668 | *M. giganteus* | -2.33 | 1.11 | SEQ | -14.33 |
| S669 | *M. giganteus* | -2.48 | -0.28 | SEQ | -14.48 |
| S670 | *M. giganteus* | -1.8 | -0.37 | SEQ | -13.8 |
| S675 | *M. giganteus* | -6.04 | 0.05 | SEQ | -18.04 |
| S676 | *M. giganteus* | -3.87 | -2.82 | SEQ | -15.87 |
| S671 | *M. giganteus* | -5.42 | 0.8 | SEQ | -17.42 |
| S672 | *M. giganteus* | -2.32 | 1.3 | SEQ | -14.32 |
| S673 | *M. giganteus* | -3 | -0.54 | SEQ | -15 |
| S674 | *M. giganteus* | -2.89 | 0.75 | SEQ | -14.89 |
| S682 | *M. giganteus* | -2.09 | 0.41 | SEQ | -14.09 |
| S617 | *M. giganteus* | -2.76 | 3.1 | BBS | -14.76 |
| S618 | NA | -5.46 | 2.57 | BBS | -17.46 |
| S619 | NA | -2.49 | 1.91 | BBS | -14.49 |
| S620 | *M. giganteus* | -2.5 | 2.15 | BBS | -14.5 |
| S621 | *M. giganteus* | -3.3 | 2.83 | BBS | -15.3 |
| S612 | *M. giganteus* | -4.62 | 1.77 | BBS | -16.62 |
| S616 | *M. giganteus* | -2.67 | 1.7 | BBS | -14.67 |
| S615 | *M. giganteus* | -4.9 | 2.34 | BBS | -16.9 |
| S611 | *M. rufogriseus* | -8.56 | 2.33 | BBS | -20.56 |
| S613 | *M. giganteus* | -2.98 | 2.08 | BBS | -14.98 |
| S614 | *M. giganteus* | -3 | 2.82 | BBS | -15 |
| S600 | *M. giganteus* | -8.99 | 1.91 | BBS | -20.99 |
| S601 | *M. giganteus* | -11 | 2.12 | BBS | -23 |
| S610 | *M. fuliginosus* | -2.99 | 4.2 | BBS | -14.99 |
| S609 | *M. giganteus* | -6.61 | 2.16 | BBS | -18.61 |
| S604 | *M. giganteus* | -6.36 | 1.84 | BBS | -18.36 |
| S602 | NA | -6.47 | 1.93 | BBS | -18.47 |
| S603 | *M. giganteus* | -5.94 | 0.84 | BBS | -17.94 |
| S608 | *M. giganteus* | -6.02 | 3.1 | BBS | -18.02 |
| S605 | NA | -8.01 | 2.88 | BBS | -20.01 |
| S607 | *M. giganteus* | -3.51 | 2.97 | BBS | -15.51 |
| S606 | *M. giganteus* | -2.88 | 2.81 | BBS | -14.88 |
| S662 | *M. giganteus* | -4.37 | 0.12 | BBS | -16.37 |
| S677 | *M. rufogriseus* | -10.43 | 1.99 | BBS | -22.43 |
| S393 | *M. antilopinus* | -1.51 | -0.42 | CYP | -13.51 |
| S392 | *M. agilis* | -7.42 | -1.4 | CYP | -19.42 |
| S394 | *M. agilis* | -7.53 | -1.05 | CYP | -19.53 |
| S395 | *M. agilis* | -11.63 | -1.14 | CYP | -23.63 |
| S391 | NA | -7.09 | 0.16 | CYP | -19.09 |
| S390 | *M. agilis* | -8.67 | -0.9 | CYP | -20.67 |
| S396 | *M. agilis* | -8.46 | 2.09 | CYP | -20.46 |
| S397 | *M. agilis* | -13.89 | 0.2 | CYP | -25.89 |
| S398 | *M. agilis* | -6.85 | -1.35 | CYP | -18.85 |
| S358 | *M. bernardus* | -8 | 0.28 | ARP | -20 |
| S359 | *M. agilis* | -8.31 | -3.4 | ARP | -20.31 |
| S290 | *M. robustus* | -6.29 | -1.95 | ARP | -18.29 |
| S295 | *M. antilopinus* | -7.99 | 0.3 | ARP | -19.99 |
| S50 | *M. agilis* | -8.82 | -3.11 | ARP | -20.82 |
| S294 | *M. bernardus* | -7.63 | -2.33 | ARP | -19.63 |
| S70 | *M. rufus* | -4.89 | 3.5 | MGD | -16.89 |
| S71 | *M. rufus* | -5.85 | 5.34 | MGD | -17.85 |
| S69 | NA | -5.46 | 2.04 | MGD | -17.46 |
| S68 | *M. robustus* | -4.92 | 1.28 | MGD | -16.92 |
| S67 | *M. rufus* | -5.72 | 5.92 | MGD | -17.72 |
| S32 | *M. rufus* | -6.99 | 4.53 | MGD | -18.99 |
| S66 | *M. rufus* | -4.58 | 4.95 | MGD | -16.58 |
| S33 | *M. rufus* | -3.35 | 7.76 | MGD | -15.35 |
| S34 | *M. rufus* | -6.94 | 6.57 | MGD | -18.94 |
| S65 | NA | -5.23 | 6.79 | MGD | -17.23 |
| S64 | *M. giganteus* | -4.23 | 4.5 | MGD | -16.23 |
| S63 | NA | -3.26 | 3.07 | MGD | -15.26 |
| S62 | NA | -5.2 | 2.67 | MGD | -17.2 |
| S61 | *M. rufus* | -4.37 | 4.22 | MGD | -16.37 |
| S60 | *M. rufus* | -5.37 | 7.46 | MGD | -17.37 |
| S59 | NA | -2.88 | 0.56 | MGD | -14.88 |
| S57 | *M. giganteus* | -5.86 | 3.71 | MGD | -17.86 |
| S58 | *M. giganteus* | -6.5 | 4.92 | MGD | -18.5 |
| S56 | NA | -2.29 | 1.77 | MGD | -14.29 |
| S17 | *M. robustus* | -7.18 | 3.48 | MGD | -19.18 |
| S43 | NA | -9.11 | 0.88 | MGD | -21.11 |
| S16 | NA | -12.07 | 0.83 | MGD | -24.07 |
| S55 | *M. rufus* | -8.12 | 5.08 | MGD | -20.12 |
| S15 | *M. rufus* | -12.62 | 1.36 | MGD | -24.62 |
| S54 | *M. rufus* | -8.86 | 2.9 | MGD | -20.86 |
| S53 | *M. giganteus* | -2.83 | 1.94 | MGD | -14.83 |
| S52 | NA | -3.67 | 0.19 | MGD | -15.67 |
| S46 | *M. rufus* | -5.84 | 3.34 | MGD | -17.84 |
| S47 | *M. rufus* | -6.52 | 2.42 | MGD | -18.52 |
| S51 | NA | -4.05 | 2.7 | MGD | -16.05 |
| S48 | *M. rufus* | -6.05 | 0.53 | MGD | -18.05 |
| S49 | NA | -5.69 | 1.61 | MGD | -17.69 |
| S478 | *M. giganteus* | -13.5 | -1.06 | SEH | -25.5 |
| S477 | *M. giganteus* | -10.82 | -1.61 | SEH | -22.82 |
| S476 | *M. giganteus* | -13.63 | -2.06 | SEH | -25.63 |
| S475 | *M. giganteus* | -11.97 | -2.66 | SEH | -23.97 |
| S474 | *M. giganteus* | -12.47 | -0.2 | SEH | -24.47 |
| S458 | *M. giganteus* | -12.52 | -2.06 | SEH | -24.52 |
| S473 | *M. giganteus* | -9.94 | -0.56 | SEH | -21.94 |
| S472 | *M. giganteus* | -9.09 | -2.59 | SEH | -21.09 |
| S459 | *M. giganteus* | -14.13 | -1.58 | SEH | -26.13 |
| S469 | *M. giganteus* | -14.68 | -2.64 | SEH | -26.68 |
| S470 | *M. giganteus* | -13.96 | -3.23 | SEH | -25.96 |
| S471 | *M. giganteus* | -13.68 | -0.22 | SEH | -25.68 |
| S465 | *M. giganteus* | -12.81 | -3.07 | SEH | -24.81 |
| S466 | *M. giganteus* | -17.16 | -2.91 | SEH | -29.16 |
| S464 | *M. giganteus* | -16.09 | -3.98 | SEH | -28.09 |
| S460 | *M. giganteus* | -15.29 | -3.1 | SEH | -27.29 |
| S461 | *M. giganteus* | -15.42 | -1.87 | SEH | -27.42 |
| S462 | *M. giganteus* | -14.96 | -2.61 | SEH | -26.96 |
| S463 | *M. giganteus* | -12.56 | -3.14 | SEH | -24.56 |
| S456 | *M. giganteus* | -17.22 | -1.78 | SEH | -29.22 |
| S457 | *M. giganteus* | -12.04 | -3.12 | SEH | -24.04 |
| S479 | *M. giganteus* | -15.52 | -2.94 | SEH | -27.52 |
| S480 | *M. giganteus* | -15.61 | -2.81 | SEH | -27.61 |
| S455 | *M. giganteus* | -12.81 | -2.62 | SEH | -24.81 |
| S468 | *M. giganteus* | -15.54 | -3.73 | SEH | -27.54 |
| S467 | *M. giganteus* | -14.66 | -2.68 | SEH | -26.66 |
| S454 | *M. giganteus* | -11.67 | -2.94 | SEH | -23.67 |
| S481 | *M. giganteus* | -16.08 | -0.25 | SEH | -28.08 |
| S482 | *M. giganteus* | -17.43 | -2.9 | SEH | -29.43 |
| S483 | *M. giganteus* | -14.59 | -1.03 | SEH | -26.59 |
| S493 | *M. giganteus* | -18.38 | -3.74 | SEH | -30.38 |
